# A Method for Estimating View Transformations from Image Correspondences Based on the Harmony Search Algorithm

**DOI:** 10.1155/2015/434263

**Published:** 2015-08-03

**Authors:** Erik Cuevas, Margarita Díaz

**Affiliations:** ^1^Departamento de Ciencias Computacionales, Universidad de Guadalajara, CUCEI , Avenida Revolución 1500, 44430 Guadalajara, JAL, Mexico; ^2^División de Ciencia y Tecnología, Universidad de Guadalajara, CU-Norte, Carretera Federal No. 23, Km. 191, 46200 Colotlán, JAL, Mexico

## Abstract

In this paper, a new method for robustly estimating multiple view relations from point correspondences is presented. The approach combines the popular random sampling consensus (RANSAC) algorithm and the evolutionary method harmony search (HS). With this combination, the proposed method adopts a different sampling strategy than RANSAC to generate putative solutions. Under the new mechanism, at each iteration, new candidate solutions are built taking into account the quality of the models generated by previous candidate solutions, rather than purely random as it is the case of RANSAC. The rules for the generation of candidate solutions (samples) are motivated by the improvisation process that occurs when a musician searches for a better state of harmony. As a result, the proposed approach can substantially reduce the number of iterations still preserving the robust capabilities of RANSAC. The method is generic and its use is illustrated by the estimation of homographies, considering synthetic and real images. Additionally, in order to demonstrate the performance of the proposed approach within a real engineering application, it is employed to solve the problem of position estimation in a humanoid robot. Experimental results validate the efficiency of the proposed method in terms of accuracy, speed, and robustness.

## 1. Introduction

The goal of estimating geometric relations in images is to find an appropriate global transformation to overlay images of the same scene taken at different viewpoints. It can be applied in image processing when an object moves in front of a static camera and when a static scene is captured by a moving camera or multiple cameras from different viewpoints. This methodology has been widely adopted in many applications, for instance, when series of images can be stitched together to generate a panorama image [1–3]. Also, multiple image superresolution approaches can be applied in the overlapped region calculated according to the estimated geometry [4–6]. The motion of a moving object can also be estimated using its geometric relations [7] and a distributed camera network can be calibrated, where each camera's position, orientation, and focal length can be calculated based on their correspondences [8–10]. Another example is the robot position that can be controlled or estimated through the estimation of the fundamental matrix/homography [11–13].

In a modelling problem, those data that can be explained by the hypothetical model are known as* inliers *of this model. Other points, for example, those generated by matching errors, are called* outliers*. The outliers are caused by external effects not related to the investigated model. Based on different criteria, several robust techniques have been proposed to identify points as inliers or outliers, being the random sampling consensus (RANSAC) algorithm [14] the most well known [15–17].

RANSAC adopts a simple hypothesize-and-evaluation process. Under such approach, a minimal subset of elements (correspondences) is sampled randomly, and a candidate model is hypothesized using this subset. Then, the candidate model is evaluated on the entire dataset separating all elements from the dataset into inliers and outliers, according to their degree of matching (error scale) to the candidate model. These steps are iterated until there is a high probability that an accurate model could be found during iterations. The model with the largest number of inliers is considered as the estimation result.

Although RANSAC algorithm is simple and powerful, it presents two main problems [18, 19]: the high consumption of iterations and the inflexible definition of its objective function. In the RANSAC algorithm, candidate models are generated by selecting data samples. Since such a strategy is completely random, a large number of iterations are required to explore a representative subset of noisy data and to find a reliable model that could contain the maximum number of inliers. In general terms, the number of iterations is strongly affected by the contamination level of the dataset. The other crucial issue is the objective function to evaluate the correctness of a candidate model from contaminated data. In the RANSAC methodology, the best estimation result is the model that maximizes the number of inliers. Therefore, the objective function involves the count, one by one, of the number of inliers associated with a candidate model. Such an objective function is fixed and prone to obtain suboptimal models under different circumstances [19].

Several variants have been proposed in order to enhance the performance of the RANSAC method. One example constitutes the approach MLESAC [20] which searches the best hypothesis by maximizing the likelihood via the RANSAC process by assuming that the inlier data would distribute as a Gaussian function and outliers are distributed randomly. Alternatively, instead of giving the error scale (i.e., the threshold to separate inliers from outliers) a priori, the SIMFIT method [21] proposes its prediction based on an iterative procedure. Other representative works, such as the projection-pursuit method [22] and TSSE (two-step scale estimator) [23], employ the mean shift technique to model the inlier distribution and obtain an inlier scale. Such approaches enables RANSAC to be data-driven; however, the whole process becomes quite time consuming.

Although all the proposed variants allow solving one of the two main RANSAC problems, the other challenge still remains. Such situation comes from the fact that the estimation process is approached as an optimization problem where the search strategy is a random walking algorithm while the objective function is fixed to the number of inliers associated with the candidate model. In order to overcome the typical RANSAC problems, we propose to visualize the RANSAC operation as a generic optimization procedure. Under this point of view, a new efficient search strategy can be added for reducing the number of consumed iterations. Likewise, it can be defined as a new objective function which incorporates other elements that allow an accurate evaluation of the quality of a candidate model.

Two important difficulties in selecting a search strategy for RANSAC are the high multimodality and the complex characteristics of the estimation process produced by the elevated contamination of the dataset. Under such circumstances, classical methods present a bad performance [24, 25], making way for recent new approaches that have been proposed to solve complex and ill-posed engineering problems. These methods include the application of modern optimization techniques such as evolutionary algorithms and metaheuristic techniques [26, 27] which have delivered better solutions over those obtained by classical methods.

The harmony search algorithm (HS) introduced by Geem et al. [28] is one example of these approaches. HS is an optimization algorithm based on the metaphor of the improvisation process that occurs when a musician searches for a better state of harmony. The HS produces a new candidate solution from all existing solutions. In HS, the solution vector is analogous to the harmony in music, and its generation schemes are analogous to musician's improvisations. With regard to other metaheuristics in the literature, HS imposes fewer mathematical prerequisites; therefore, it can be easily modified for solving several sorts of engineering optimization challenges [29, 30]. Numerical comparisons have established that the convergence of HS is faster than GA [29, 31, 32]. Such a fact has attracted the attention of the evolutionary computation community. It has been effectively applied to solve a wide range of practical optimization problems such as structural optimization [33], parameter estimation of the nonlinear Muskingum model [34], design optimization of water distribution networks [35], vehicle routing [36], image segmentation [37], and circle detection in images [38].

Although HS allows identifying promising regions at the solution space within a reasonable time interval, it underperforms in local searching, in particular for parameter identification applications [39–42]. In order to enhance the fine-tuning (accuracy) properties of HS, the local search parameter (BW) is dynamically adjusted to improve the balance between exploration and exploitation during the search process (see [29]). However, considering that the adjustment follows an exponential function, longer exploitation periods are allowed, affecting the exploring capacity of HS particularly when it is applied to complex objective functions. A better adjustment alternative, which employs the use of a linear model, has been recently proposed in [43]. It presents better searching capacities than the approaches based on exponential functions. For this reason, such an approach is used in our method.

In this paper, a new method is presented for the robust estimation of multiple view relations from point correspondences. The approach combines the RANSAC method with the HS. Upon such combination, the proposed method adopts a different sampling strategy in comparison to RANSAC to generate putative solutions. Under the new mechanism, new candidate solutions are built iteratively by considering the quality of models generated by previous candidate solutions, rather than relying over a pure random selection as it is the case of RANSAC. Likewise, a more accurate objective function is incorporated to accurately evaluate the quality of a candidate model. As a result, the proposed approach can substantially reduce the number of iterations still preserving the robust capabilities of RANSAC. The method is generic and its use is illustrated by the estimation of homographies, considering synthetic and real images. Additionally, in order to demonstrate the performance of the proposed approach in a real engineering application, it is employed to solve the problem of position estimation of a humanoid robot. Experimental results validate the efficiency of the proposed method in terms of accuracy, speed, and robustness.

The paper is organized as follows.  Section 2 explains the problem of image matching considering multiple views.  Section 3 introduces the fundamentals of the RANSAC method.  Section 4 explains the harmony search algorithm while Section 5 presents the proposed approach.  Section 6 exhibits the experimental set and its performance results.  Section 7 exposes a robotic application of the proposed approach. Finally, Section 8 establishes final conclusions.

## 2. View Relations from Point Correspondences

The problem of image matching consists in finding a geometric transformation that maps one image of a scene to another image taken from a different point of view. To determine the correspondence among points, it is necessary to find corresponding points on both images. Such point pairs can be obtained as a result of applying an automatic algorithm of detection and matching [44, 45]. The detected points are described by vectors of parameters (descriptors), and frequently these parameters do not allow discriminating one point from another with complete certainty. As a result, an erroneous matching about the correspondence of points located on different parts of different images may emerge.

In this section the geometric relations of points between two views are discussed, considering the case of homography.

Assume that there is a collection of pairs of the corresponding points that are found on two images

(1) $$\begin{array}{c} U=\left\{\left(x_{1},x_{1}^{\prime }\right),\left(x_{2},x_{2}^{\prime }\right),\ldots ,\left(x_{M},x_{M}^{\prime }\right)\right\}, \end{array}$$

where **x**_*i*_ = (*x*_*i*_, *y*_*i*_, 1)^*T*^ and **x**_*i*_′ = (*x*_*i*_′, *y*_*i*_′, 1)^*T*^ are the positions of points in the first and second images, respectively.

**Figure 1:**
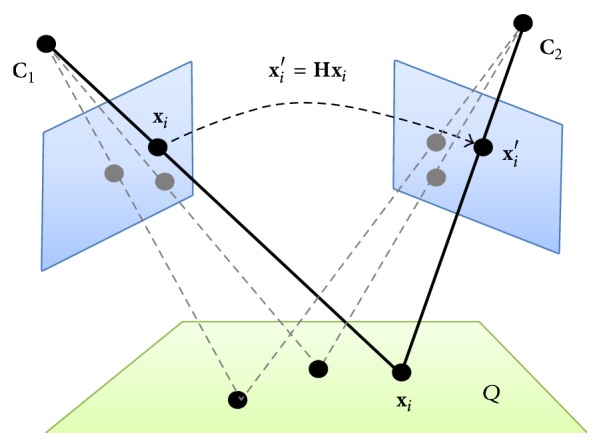
Homography from a plane between two views.

Two perspective images can geometrically be linked through a plane *Q* of the scene by a homography **H** ∈ *R* ^3×3^ (see Figure 1). This projective transformation **H** relates corresponding points of the plane projected into two images by **x**_*i*_′ = **H** **x**_*i*_ or **x**_*i*_ = **H** ^−1^ **x**_*i*_′. The homography across two views can be computed by solving a linear system from a set of four point matches [46]. The quality of the estimated homography **H** is evaluated by considering the distance between the position of the point calculated with the help of the matrix **H** and the actually observed position. Therefore, the mismatch error *EH*_*i*_^2^ produced by the *i*-correspondence (**x**_*i*_, **x**_*i*_′) is defined as the sum of squared distances from the points to their estimated positions:

(2) $$\begin{array}{c} EH_{i}^{2}={\left[d\left(x_{i}^{\prime },Hx_{i}\right)\right]}^{2}+{\left[d\left(x_{i},H^{-1}x_{i}^{\prime }\right)\right]}^{2}, \end{array}$$

where *η* = *d*(**x**_*i*_, **H** ^−1^ **x**_*i*_′) and *η*′ = *d*(**x**_*i*_′, **H** **x**_*i*_) correspond to the errors produced in the first and second images, respectively.

**Figure 2:**
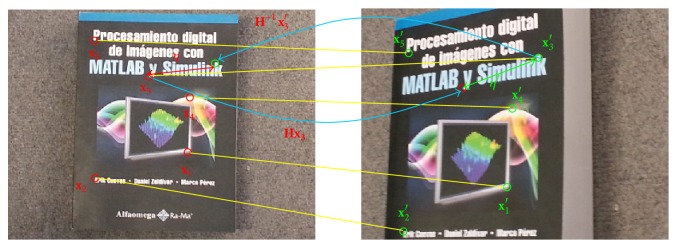
Example of evaluation process for a particular homography **H**.

Figure 2 shows the error evaluation process of **H** for a particular example which involves five correspondences **U** = {(**x**_1_, **x**_1_′),…, (**x**_5_, **x**_5_′)} distributed in both views. In the example, the correspondence (**x**_3_, **x**_3_′) presents a considerable error evaluated as the distances (*η*, *η*′) between the points (**x**_3_, **x**_3_′) and their positions calculated with the use of the matrix **H**  (**H** **x**_3_, **H** ^−1^ **x**_3_′).

## 3. Random Sampling Consensus (RANSAC) Algorithm

The goal of RANSAC is to estimate the geometric transformation (the homography **H**) from image correspondences over two views. Potentially there are a significant number of mismatches amongst the correspondences. Correct matches will obey the homography transformation. Therefore, the aim is to obtain a set of* inliers* consistent with the homography transformation by using a robust technique. In this case* outliers* are points inconsistent with the homography transformation. In order to solve such a problem, the RANSAC algorithm has proven to be the most successful [15–17].

RANSAC solves the problem of model parameters estimation by finding the best hypothesis *h* ^*B*^ among the set of all possible hypotheses *H* generated by the source data. Such source data are typically contaminated by noise. In order to build the hypothesis *h*_*i*_ about the unknown parameters, a sample **S**_*i*_ of the minimum size (*s*) required for model estimation is obtained (e.g., a sample of only two points is sufficient to calculate a straight line, *s* = 2, and of four to obtain a homography, *s* = 4). Under this consideration, the probability of finding an outlier is reduced. Considering that the number of elements contained in a sample is small, the amount of possible samples that can be generated from the complete source data **U** is enormous. Under such circumstances, the exhausting testing of all samples for a reasonable time is impossible. RANSAC faces such problem because it only considers *G* samples which are randomly selected and evaluated. Algorithms of the RANSAC family consist of *G* iterations of the following cycle.

(1) Construct a sample **S**_*i*_ ⊂ **U** consisting of *s* different elements.
(2) Build the hypothesis *h*_*i*_ based on the sample **S**_*i*_.
(3) Evaluate the degree of agreement *A*_*i*_ of the hypothesis *h*_*i*_ with the set of all source data **U**.

After the construction and evaluation of all *G* hypotheses, the hypothesis *h* ^*B*^ with the best degree of agreement is chosen among them. It is considered as a robust estimate of the model parameters. Such operation can be described as follows:

(3) $$\begin{array}{c} h^{B}=\underset{i=1,\ldots ,G}{\arg\;\max}\;A_{i}\left(U,h_{i}\right). \end{array}$$

The maximization of the degree of agreement (number of inliers) is equivalent to the minimization of the penalty function whose value depends on the number of outliers. Therefore, the degree of agreement *A*_*i*_(**U**, *h*_*i*_) is computed as follows:

(4) $$\begin{array}{c} A_{i}\left(U,h_{i}\right)=\overset{M}{\underset{j=1}{\sum }}\theta \left(e_{j}^{2}\left(h_{i}\right)\right),\;j=1,\ldots ,M,\\ \theta \left(e_{j}^{2}\left(h_{i}\right)\right)=\left\{\begin{array}{cc} 0 & e_{j}^{2}\left(h_{i}\right)>\text{Th}\\ 1 & e_{j}^{2}\left(h_{i}\right)\leq \text{Th}, \end{array}\right. \end{array}$$

where Th is a permissible error, *M* is the number of elements contained in the source data **U**, and *e*_*j*_^2^(*h*_*i*_) is the quadratic error produced by the *j*th data considering the hypothesis *h*_*i*_. In the context of this paper, *e*_*j*_^2^(*h*_*i*_) corresponds to *EH*_*i*_^2^ which represents the error produced by the *i*th correspondence.

The hypothesis with a minimum penalty (i.e., with the maximum degree of agreement) is chosen as the best matching criterion. In the original scheme of RANSAC, the quality of a hypothesis is defined as the number of inliers. For a given value of the permissible error Th, the point *j* that produces the error *e*_*j*_^2^(*h*_*i*_) is regarded to be an inlier of *h*_*i*_ if its value does not exceed the threshold Th; otherwise the point is regarded as an outlier.

In the RANSAC algorithm, the optimal hypothesis *h* ^*B*^ is found and the penalty is minimized by using a search strategy of random walking; therefore many attempts are necessary to investigate in sufficient detail the space of possible samples and to find the sample for which the hypothesis has the greatest degree of agreement on the source data. The number of iterations and thus the time spent for the search can be reduced by choosing points according to some directed rules, rather than randomly. Optimization algorithms can be considered as a robust scheme in contrast to the random search [47]. In an optimization algorithm, new candidate solutions are generated in accordance to the information obtained from past candidate solutions.

In this paper, we propose a different approach based on the HS as optimization algorithm. The goal is to demonstrate that the new method, by combining the idea of testing minimum-sized samples with the directed search inspired by the improvisation process that occurs when a musician searches for a better state of harmony, allows performing an efficient search among the correspondences to generate models of higher quality. It is also shown that the number of inliers found by the new method with the use of a fixed number of samples is significantly greater than the number of inliers determined by the family of algorithms based on RANSAC.

## 4. Harmony Search Algorithm

In the basic HS, each solution is called a “harmony” and is represented by an *n*-dimension real vector. An initial population of harmony vectors are randomly generated and stored within a harmony memory (HM). A new candidate harmony is thus generated from the elements in the HM by using a memory consideration operation either by a random reinitialization or a pitch adjustment operation. Finally, the HM is updated by comparing the new candidate harmony and the worst harmony vector in the HM. The worst harmony vector is replaced by the new candidate vector in case it is better than the worst harmony vector in the HM. The above process is repeated until a certain termination criterion is met. The basic HS algorithm consists of three basic phases: HM initialization, improvisation of new harmony vectors, and updating of the HM. The following discussion addresses details about each stage.

### 4.1. Initializing the Problem and Algorithm Parameters

In general, the global optimization problem can be summarized as follows: min *f*(**p**) : *p*(*j*) ∈ [*l*(*j*), *u*(*j*)], *j* = 1,2,…, *n*, where *f*(**p**) is the objective function, **p** = (*p*(1), *p*(2),…, *p*(*n*)) is the set of design variables, *n* is the number of design variables, and *l*(*j*) and *u*(*j*) are the lower and upper bounds for the design variable *p*(*j*), respectively. The parameters for HS are the harmony memory size, that is, the number of solution vectors lying on the harmony memory (HM), the harmony-memory consideration rate (HMCR), the pitch adjusting rate (PAR), the distance bandwidth (BW), and the number of improvisations (NI) which represents the total number of iterations. It is obvious that an adequate selection for HS parameters would enhance the algorithm's ability to search for the global optimum under a high convergence rate.

### 4.2. Harmony Memory Initialization

In this stage, initial vector components at HM, that is, HMS vectors, are configured. Let **p**_*i*_ = {*p*_*i*_(1), *p*_*i*_(2),…, *p*_*i*_(*n*)} represent the *i*th randomly generated harmony vector: *p*_*i*_(*j*) = *l*(*j*)+(*u*(*j*) − *l*(*j*)) · rand(0,1) for *j* = 1,2,…, *n* and *i* = 1,2,…, HMS, where rand(0,1) is a uniform random number between 0 and 1. Then, the HM matrix is filled with the HMS harmony vectors as follows:

(5) $$\begin{array}{c} \text{HM}=\left[\begin{array}{c} p_{1}\\ p_{2}\\ \vdots \\ p_{\text{HMS}} \end{array}\right]. \end{array}$$

### 4.3. Improvisation of New Harmony Vectors

In this phase, a new harmony vector **p**_new_ is built by applying the following three operators: memory consideration, random reinitialization, and pitch adjustment. Generating a new harmony is known as “improvisation.” In the memory consideration step, the value of the first decision variable *p*_new_(1) for the new vector is chosen randomly from any of the values already existing in the current HM, that is, from the set {*p*_1_(1), *p*_2_(1),…, *p*_HMS_(1)}. For this operation, a uniform random number *r*_1_ is generated within the range [0, 1]. If *r*_1_ is less than HMCR, the decision variable *p*_new_(1) is generated through memory considerations; otherwise, *p*_new_(1) is obtained from a random reinitialization between the search bounds [*l*(1), *u*(1)]. Values of the other decision variables *p*_new_(2), *p*_new_(3),…, *p*_new_(*n*) are also chosen accordingly. Therefore, both operations, memory consideration and random reinitialization, can be modelled as follows:

(6) pnew(j)=pi(j)∈p1(j),p2(j),…,pHMS(j)kkkkkkkwith  probability  HMCRl(j)+(u(j)−l(j))·rand(0,1)kkwith  probability  1−HMCR.

Every component obtained by memory consideration is further examined to determine whether it should be pitch-adjusted. For this operation, the pitch adjusting rate (PAR) is defined as to assign the frequency of the adjustment and the bandwidth factor (BW) to control the local search around the selected elements of the HM. Hence, the pitch adjusting decision is calculated as follows:

(7) pnewj=pnew(j)=pnew(j)±rand(0,1)·BWpnew(j)ggggggwith  probability  PARpnewj with  probability  1−PAR.

Pitch adjusting is responsible for generating new potential harmonies by slightly modifying original variable positions. Such operation can be considered similar to the mutation process in evolutionary algorithms. Therefore, the decision variable is either perturbed by a random number between −BW and BW or left unaltered. In order to protect the pitch adjusting operation, it is important to assure that points lying outside the feasible range [*l*, *u*] must be reassigned, that is, truncated to the maximum or minimum value of the interval.

### 4.4. Updating the Harmony Memory

After a new harmony vector **p**_new_ is generated, the harmony memory is updated by the survival of the fit competition between **p**_new_ and the worst harmony vector **p**_*w*_, according to its fitness value, in the HM. Therefore **p**_new_ will replace **p**_*w*_ and become a new member of the HM in case the fitness value of **p**_new_ is better than the fitness value of **p**_*w*_.

### 4.5. Computational Procedure

The computational procedure of the basic HS can be summarized as shown in Procedure 1 [18].

### 4.6. Dynamical Linear Adjustment of BW

Every metaheuristic algorithm needs to address the issue of exploration-exploitation of the search space. Exploration is the process of visiting entirely new points of a search space whilst exploitation is the process of refining those points within the neighborhood of previously visited locations in order to improve their solution quality.

In HS, the BW parameter controls the local search around HM elements. A large BW value eases the algorithm's searching at a larger scope, while a small BW value is appropriate for fine-tuning of best solution vectors.

In the standard HS, the BW value is considered as a constant number. However, in this work, the BW value is dynamically adjusted as to favor exploration at early stages while exploitation is reinforced during final stages of the searching process. The adjustment uses a linear model defined as follows:

(8) BWk=BWmax⁡−BWmax⁡−BWmin⁡2·NI·3kif  k<23NI
BWmin⁡if  k≥23NI,

where *k* is the iteration index, while BW_max⁡_ and BW_min⁡_ are the maximum and minimum BW values, respectively. In contrast to exponential adjustment [26], linear models, as the one used in this paper, allow a better balance between exploration and exploitation (fine-tuning) of the search process [40].

Since all candidate solutions are generated by using the HS operators, there is a low probability to be trapped into local minima [48]. HS can effectively handle challenging multimodal optimization problems [49, 50]. Such fact contrasts to well-known genetic algorithms (GA) [51] and particle swarm optimization (PSO) [52] which usually tends to conduct the whole population towards the best candidate solution [53] producing premature convergence.

## 5. Method for Geometric Estimation Using HS

The estimation of model parameters in algorithms of the RANSAC family is implied to find an optimal sample of length *s* from a set consisting of *M* elements. In the standard scheme, RANSAC uses a random walking algorithm as a search strategy. The idea of the proposed method considers the use of HS to generate samples based on information about their quality, rather than randomness. The quality of a sample, that is, the fitness of a harmony *f*(**p**_*i*_), is defined as the matching degree of the hypothesis *h*_*i*_ that is constructed based on the correspondence numbers coded within **p**_*i*_.

Considering that the problem consists in estimating the parameters of **H** through a set **U** = {(**x**_1_, **x**_1_′), (**x**_2_, **x**_2_′),…, (**x**_*M*_, **x**_*M*_′)} of *M* different correspondences, the proposed approach can be described as shown in Algorithm 1.

The proposed approach combines the RANSAC method with the HS adopting a different sampling strategy in comparison to RANSAC to generate putative solutions. Under the new mechanism, at each iteration, new candidate solutions are built taking into account the quality of the models that have been generated by previous candidate solutions, rather than purely random as it is the case in RANSAC.

Since the approach visualizes the RANSAC operation as a generic optimization procedure, different objective functions can be incorporated to accurately evaluate the quality of a candidate model. Although several objective functions can be tested, this work employs the expression in Equation (A).

In contrast to the traditional RANSAC algorithm, the objective function considers two different aims: the number of inliers and the approximation error. The idea is to find the candidate homography that maximizes the number of inliers and simultaneously minimizes the approximation error. Under such circumstances, the obtained estimation represents the solution that presents the best trade-off between both objectives. As a result, the proposed approach can substantially reduce the number of iterations, still preserving the robust capabilities of RANSAC method.

## 6. Experimental Results

In this section, a comprehensive set of experiments have been conducted to test the performance of the proposed approach. The results are divided into two different categories: (1) effect of the main HS parameters in the estimation results and (2) comparison results over synthetic and real homographies.

In the experiments, three performance indexes are considered: the number of inliers (NofI), the error (*E*_*s*_, *E*_*r*_), and the number of function evaluations (NFE). The first two indexes assess the accuracy of the solution whereas the last one measures the computational cost.

The number of inliers (NofI) expresses the amount of elements contained in the set **I** of detected inliers. The error (*E*_*s*_, *E*_*r*_) provides a quality measure of the estimated relation. In case of synthetic data, the error is calculated as

(9) $$\begin{array}{c} E_{s}={\left(\underset{ij}{\sum }\frac{d^{2}(x_{i}^{j},{\hat{x}}_{i}^{j})}{\text{NofI}}\right)}^{1/2},\;i\in I,\;\;j\in \left\{1,2\right\}, \end{array}$$

where **x**_*i*_^*j*^ is the inlier point calculated by the estimated relation in the *j*-view, ${\hat{x}}_{i}^{j}$ is the inlier ground true point, and *d*(·) is the Euclidian distance between the points. Therefore, *E*_*s*_ evaluates the fit of the estimated relation, computed from the noisy data, against the known ground truth points.

In the case of real data the error is assessed from the standard deviation of the inliers. Thus, *E*_*r*_ is computed as follows:

(10) $$\begin{array}{c} E_{r}={\left(\underset{i}{\sum }\frac{e_{i}^{2}}{\text{NofI}}\right)}^{1/2},\;i\in I, \end{array}$$

where *e*_*i*_^2^ is the quadratic error produced by the *i*th inlier. In the context of this paper, *e*_*i*_^2^ corresponds to *EH*_*i*_^2^ which represents the error produced by the *i*th inlier.

The number of function evaluations (NFE) specifies the total number of transformations that have been evaluated by the algorithm until the best estimation has been reached.

### 6.1. Effect of the HS Parameters

**Figure 3:**
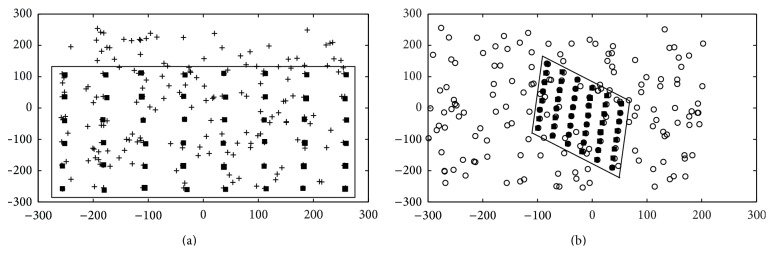
A test example where the HS-RANSAC has been applied to estimate a random transformation **H** considering only the 75% of additional outliers. (a) The first view and (b) the second view, with black squares representing the detected inliers.

Several parameters define the performance of HS. However, from all of them, the harmony-memory consideration rate (HMCR) and pitch adjusting rate (PAR) are the most important [55]. To study the impact of these parameters, over the performance of HS in the estimation procedure, different values have been tested on the computation of a synthetic homography. Such a homography was generated, in its first view, by using a rectangular pattern of 8 × 6 elements within a 2-dimensional space of [−300,300]. Then, such points were transformed by a random homography **H** and contaminated by normally distributed noise for constructing their correspondences in the second view. A set of outliers was added by selecting randomly data points within the space limits. In the test, the fraction of outliers is of 75%. In order to illustrate the experimental setup, Figures 3(a) and 3(b) exhibit the first and second views, respectively. Considering the correspondence points, the HS-RANSAC algorithm generates the estimation of **H**. In Figure 3(a), the black squares indicate the position in the first view of a point from the second view as a result of the **H** transformation. Likewise, the black squares in Figure 3(b) exhibit the position in the second view of a point from the first view as a result of the **H** transformation.

In the experiment, the maximum number of iterations is set to 950. HMS, BW_max⁡_, BW_min⁡_, *λ*, and Th are fixed to 50, 10, 1, 0.001, and 5, respectively. The results report the number of inliers (NofI) and the produced estimation error (*E*_*s*_) of HS-RANSAC, averaged over 30 runs, for the different values of HMCR and PAR. In the experiment, the parameter values are modified considering specific interval. HMCR varies from 0.5 to 0.8 whereas PAR changes from 0.1 to 0.4. The results, shown in Table 1, suggest that a proper combination of different parameter values can improve the performance of HS-RANSAC and the quality of the estimations. The best parameter configuration in the experiment is highlighted in Table 1.

After considering the analysis of Table 1, the parameter values for the proposed estimator are defined in Table 2. Once defined, such values have been kept in all experiments reported in this paper.

### 6.2. Comparison Results over Synthetic and Real Homographies

We have applied the proposed method to estimate homographies on real and synthetic data in order to compare its performance against other estimation algorithms such as the standard RANSAC [14], the MLESAC [20], the SIMFIT method [21], the projection-pursuit algorithm [22], the TSSE [23], and the PSO algorithm (PSO-RANSAC) [54]. The first five approaches are RANSAC-based estimators whose results are broadly known. In all cases, the algorithms are tuned according to the value set which is originally proposed by their own references. However, the PSO method has been included as a reference, only to validate the performance of the HS as an optimization approach.

In order to conduct a fair comparison between the HS version used in this work and PSO, an enhanced version of PSO has been also chosen with similar characteristics. Therefore, it is used in the comparisons, the PSO version reported in [54]. Such an approach is proposed to mitigate the premature convergence problem of the original PSO method. It incorporates two new elements: (1) a weight factor *w* and (2) a constriction factor *V*_max⁡_. Similar to BW in the HS method, the weight factor *w* is linearly decreased during the algorithm execution to regulate the attraction force towards the best particle seen so far. On the other hand, the constriction factor *V*_max⁡_ permits limiting the particle velocities in order to control their trajectories. Under such circumstances, the enhanced PSO version is used in combination with RANSAC considering the following configuration: *P* = 10, *c*_1_ = 2, *c*_2_ = 2, and Th = 5 whereas the weight factor *w* decreases linearly from 0.9 to 0.2. Additionally, the constriction factor *V*_max⁡_ is fixed to 2. Such a configuration presents the best possible performance according to [54].

#### 6.2.1. Homography Estimation with Synthetic Data

This section reports the experimental results corresponding to the estimation of homography matrix considering synthetic data. In the experiments, the same synthetic homography produced in Section 4.1 has been used (see Figure 3). The only difference is that the fraction of the incorporated outliers varies from 0 to 100%.

In the experiment, each algorithm's execution requires 1000 iterations. Since the proposed HS-RANSAC involves 50 initial evaluations (size of the harmony memory), it requires the execution of only 950 iterations to reach the 1000 evaluations. On the other hand, PSO-RANSAC possesses 10 particles; for this reason 100 generations need to be evolved in order to fulfill the 1000 iterations.

**Figure 4:**
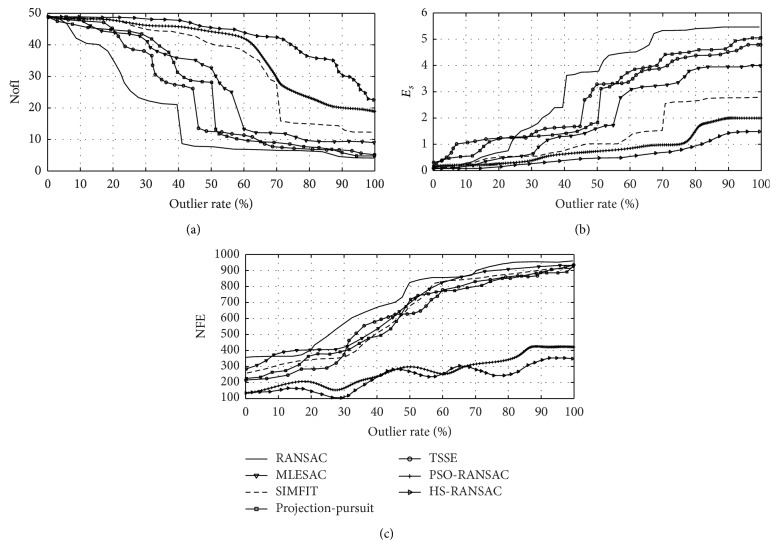
Experimental results corresponding to the estimation of **H** considering synthetic data.

Figure 4 presents the performance for each algorithm. The results present the averaged outcomes obtained throughout 50 different executions. In order to appropriately analyze these results, it is necessary to define the concept of a breakdown point [18]. The breakdown point is identified as the highest outlier ratio from which the algorithm degrades its capacity to find inliers. It can be seen from Figure 4(a) that standard RANSAC has a breakdown point at 40%, the MLESAC at 55%, the SIMFIT method at 70%, the projection-pursuit algorithm at 50%, the TSSE at 45%, and the PSO-RANSAC at 80%. In contrast to such methods, the proposed approach, HS-RANSAC, does not seem to have a prominent breakdown point, since its capacity to detect inliers smoothly degrades. It is also observed that the HS-RANSAC algorithm presents the best performance in terms of the number of inliers (NofI), as it is able to detect most of them. For the estimated **H**, the error *E*_*s*_ (Figure 4(b)) is fairly comparable for all methods until they reach their breakdown points. Nonetheless, the proposed algorithm performed better, being the only algorithm that consistently found the minimum error at all outlier ratios.

In terms of number of function evaluations (NFE), Figure 4(c) shows that the standard RANSAC, the MLESAC, the projection-pursuit algorithm, and the TSSE invest approximately the same number of iterations for reaching their best estimation of **H**. Since such methods use a random walking algorithm as a search strategy, the NFE significantly grows as the number of outliers increases. On the other hand, the PSO-RANSAC and the HS-RANSAC (that use an optimization algorithm as search strategy) maintain a considerably low NFE value with independence of the number of outliers.

From the experiment, it is evident that the use of an optimization approach can considerably reduce the NFE value. However, there is no optimization algorithm suitable to find a good enough estimation considering the high multimodality and complex characteristics of the estimation process which is produced by the elevated contamination of the dataset. Therefore, although the PSO-RANSAC finds its best estimated fundamental matrix **H** investing approximately the same number of evaluations as the HS-RANSAC, such estimated matrix represents only a suboptimal solution. This fact can be observed in Figure 4(b) where it is clear that the PSO-RANSAC algorithm presents higher *E*_*s*_ values in comparison to the HS-RANSAC approach. The reason for this problem points to those operators used by PSO for modifying the individual positions. In PSO, during their evolution, the position of each agent in the next iteration is updated yielding an attraction towards the position of the best particle seen so far. Such behavior shows that the entire population, as the algorithm evolves, concentrates around the best particle, favoring the premature convergence (reaching suboptimal solutions) [53].

#### 6.2.2. Homography Estimation with Real Images

**Figure 5:**
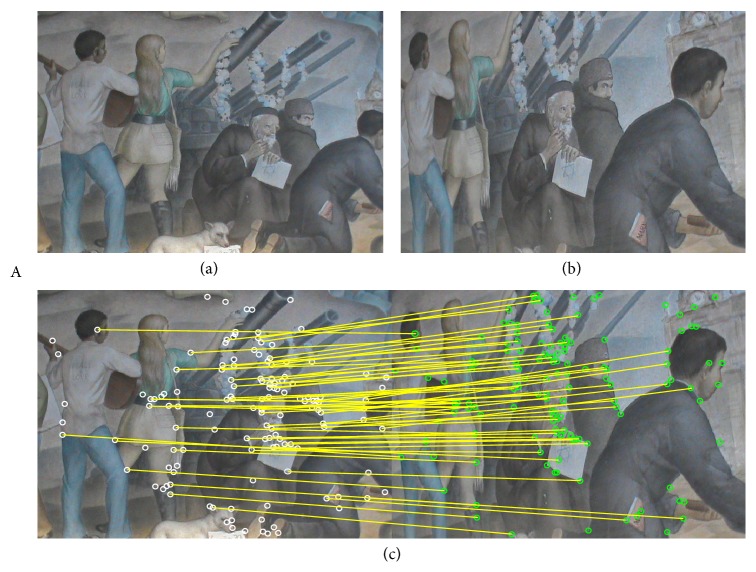
Test image “A”: (a) first view, (b) second view, and (c) correspondence points and inliers produced by HS-RANSAC.

**Figure 6:**
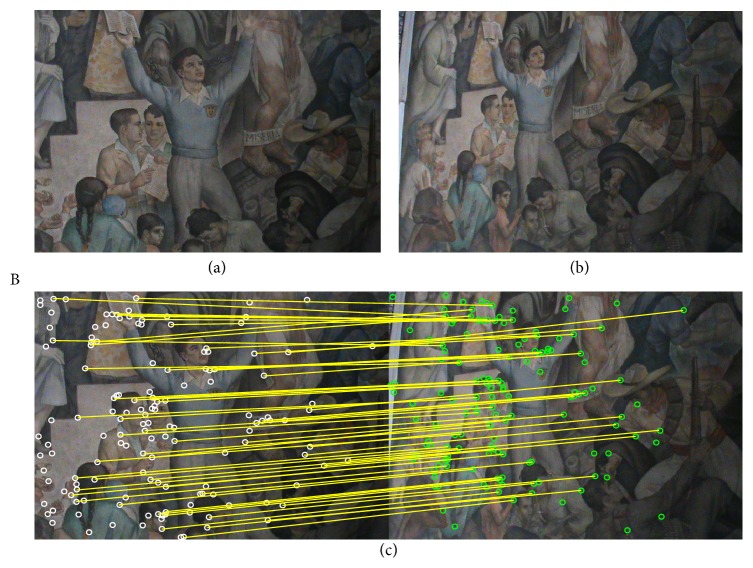
Test image “B”: (a) first view, (b) second view, and (c) correspondence points and inliers produced by HS-RANSAC.

**Figure 7:**
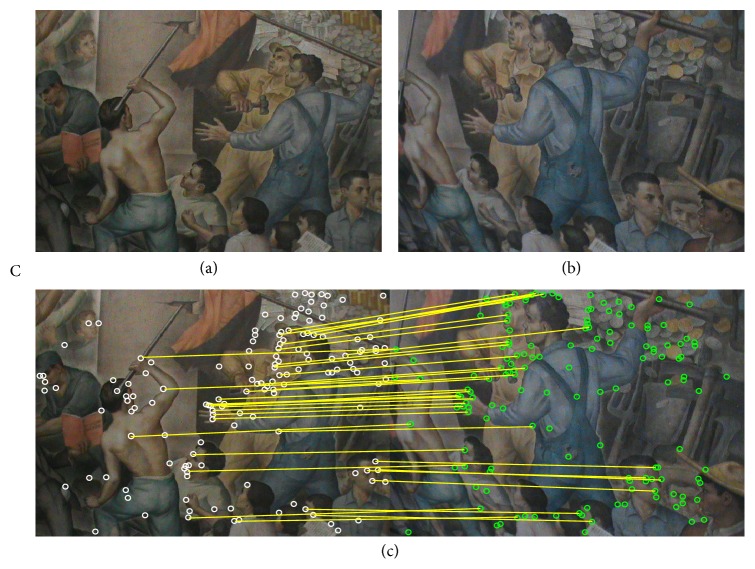
Test image “C”: (a) first view, (b) second view, and (c) correspondence points and inliers produced by HS-RANSAC.

**Figure 8:**
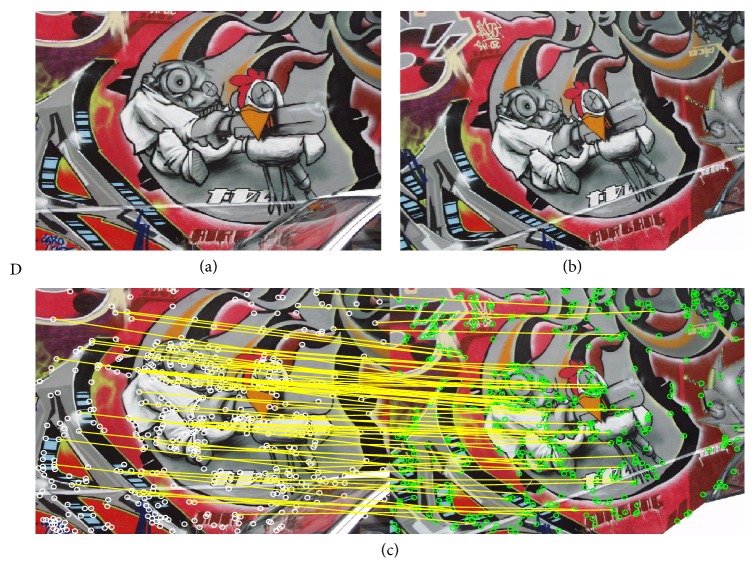
Test image “D”: (a) first view, (b) second view, and (c) correspondence points and inliers produced by HS-RANSAC.

In this section, the experimental results of the estimation of homographies **H** considering real images are reported. To evaluate the estimation performance of the proposed method, Table 3 tabulates the comparative inlier detection performance of the standard RANSAC [14], the MLESAC [20], the SIMFIT method [21], the projection-pursuit algorithm [22], the TSSE [23], the PSO algorithm (PSO-RANSAC) [54], and the proposed HS-RANSAC approach, in terms of the detection rate (DR), the error (*E*_*r*_), and the number of function evaluations (NFE). The experimental dataset includes 4 images (images A, B, C, and D) which are shown in Figures 5, 6, 7, and 8. Such images contain a determined number of inliers which have been detected and counted by a human expert (A = 86, B = 72, C = 56, and D = 122). Such values act as ground truth for all the experiments. For the comparison, the detection rate (DR) is defined as the ratio between the number of inliers correctly detected by the algorithm (NofI value) and the total number of inliers determined by the expert. The results consider 50 different executions for each algorithm over the four images. Experimental results show that the proposed HS method accomplishes at least a 94.2% of inlier detection accuracy. A close inspection of Table 3 also reveals that the proposed approach is able to achieve the smallest error (*E*_*r*_), yet requiring a few number of function evaluations (NFE) for most cases.

Figures 5, 6, 7, and 8 also exhibit the results after applying the HS-RANSAC estimator. Such results present the median case obtained throughout 50 runs.

## 7. Engineering Application: Position Estimation in a Humanoid Robot

Additionally, in order to demonstrate the performance of the proposed approach in a real engineering application, the paper also reports the application of the HS-RANSAC to solve the problem of position estimation of a humanoid robot.

In the last decades, much work has already been accomplished in the area of humanoid robotics [56, 57]. Position determination for humanoid robots is a critical problem, since it is used to control their balance and locomotion. Recently, a notable research [58] has been devoted to achieving better performance in system position for humanoid robots by using sensor fusion methods. In general, integrating information from different sensors increases not only the versatility of the system, but also its cost and complexity. Vision is one of the most studied sensory modalities for position and navigation purposes since it provides rich information of the environment.

**Figure 9:**
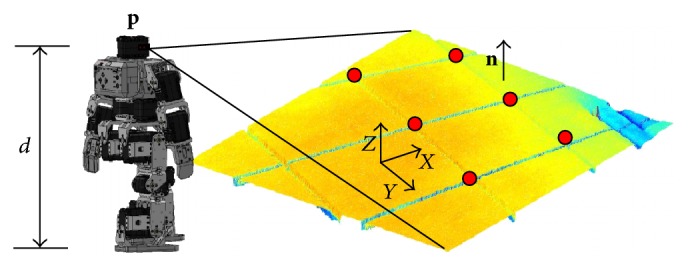
Process of planar motion calculation based on homographies.

The framework of the approach presented in this section, as an application, is a vision system consisting of a fixed camera mounted on a Bioloid© humanoid robot. In the approach, the position (*x*, *y*) of the robot is computed considering the homography estimated by the HS-RANSAC. Therefore, the idea is to calculate the planar motion of the humanoid robot through the estimated homographies.  Figure 9 illustrates the process of planar motion calculation.

The homography can be related to camera motion and plane location as follows:

(11) $$\begin{array}{c} H=R+\frac{1}{d}t^{T}n, \end{array}$$

where *d* is the distance from the camera to the plane (the height of the humanoid approximately). **R** describes a rotation *γ* about the *Z* axis and can be expressed as

(12) $$\begin{array}{c} R=\left[\begin{array}{ccc} \cos\gamma & \sin\gamma & 0\\ -\sin\gamma & \cos\gamma & 0\\ 0 & 0 & 1 \end{array}\right]. \end{array}$$

And **t** is a translation vector with the form

(13) $$\begin{array}{c} t=\left(t_{x},t_{y},0\right). \end{array}$$

As the unit normal **n** is (0,0, 1), considering the point **p**, the rotation matrix **R**, and the vector **t** (where **R** and **t** are calculated from the homography **H**), the new planar position **p**_new_ can be computed as

(14) $$\begin{array}{c} p_{\text{new}}=Rp+t. \end{array}$$

**Figure 10:**
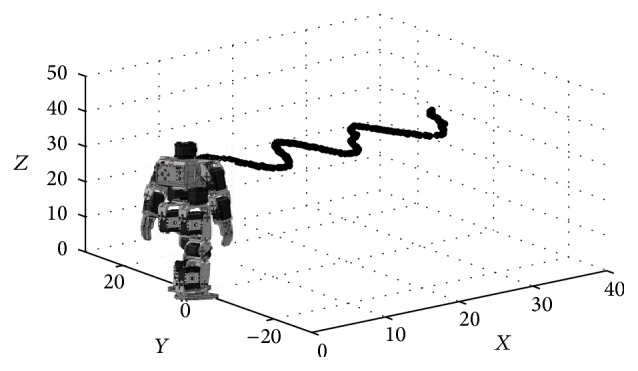
Position calculated from the homography.

More details about planar motion based on homography can be found in [59]. The HS-RANSAC algorithm and 11–14 were implemented in a Raspberry Pi. Since the computation must be verified in real time, the number of iterations is fixed to only 150.  Figure 10 shows the calculated positions from the homographies estimated during the humanoid locomotion. Such a figure demonstrates that the information of the estimated position adequately reflects the humanoid movement in spite of the reduced number of iterations.

## 8. Conclusions

In this paper, a new method for robustly estimating homographies from point correspondences based on the evolutionary algorithm has been presented. The approach combines the RANSAC method and the harmony search (HS) algorithm. With the combination, the proposed method adopts an alternative sampling strategy in comparison with RANSAC to build putative solutions. Under the new mechanism, new candidate solutions are generated iteratively by taking into consideration the quality of models produced by previous candidate solutions, instead of relying over a pure random selection as it is the case of RANSAC. On the other hand, a more accurate objective function was incorporated to adequately asses the quality of a candidate model. As a result, the proposed approach can substantially reduce the number of iterations still preserving the robust capabilities of RANSAC.

The proposed approach has been compared to other similar techniques proposed in the literature such as standard RANSAC [14], the MLESAC [17], the SIMFIT method [18], the projection-pursuit algorithm [19], the TSSE [20], and the PSO algorithm (PSO-RANSAC) [52]. The efficiency of the algorithm has been evaluated in terms of the detection rate (DR, NofI), accuracy (*E*_*s*_, *E*_*r*_), and computational cost (NFE). Experimental results that consider real and synthetic data provide evidence on the remarkable performance of the proposed algorithm in comparison to such methods. Additionally, in order to demonstrate the performance of the proposed approach in a real engineering application, it has been employed to solve the problem of position estimation in a humanoid robot.

Although the experimental results indicate that the proposed method can yield better results on estimating homographies, it should be noticed that the aim of our paper is not intended to beat all the RANSAC methods which have been proposed earlier but to show that the use of evolutionary approaches can effectively serve as an attractive alternative to solve complex optimization problems, yet demanding fewer function evaluations.

## Figures and Tables

**Algorithm 1:**
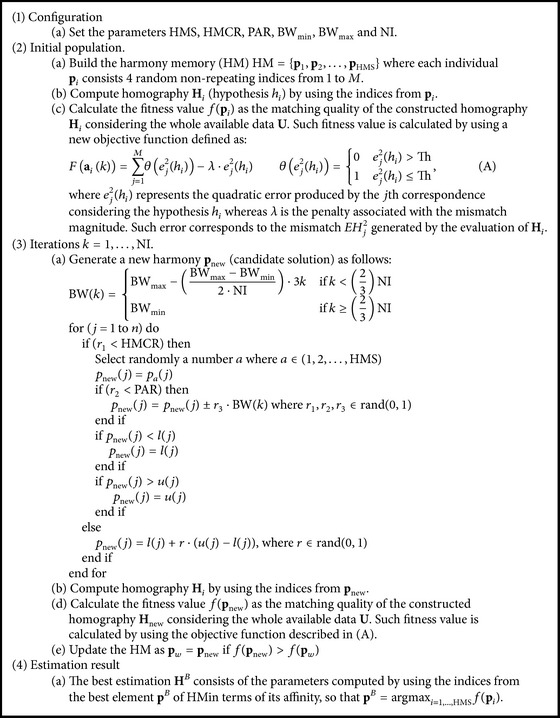


**Procedure 1:**
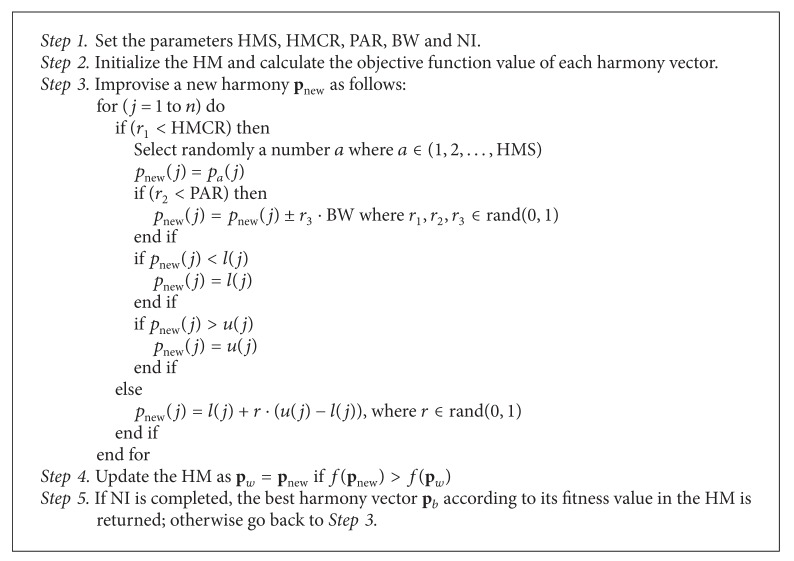


**Table 1:** Effect of the HS parameters in the estimation process.

	(NofI, *E*_*s*_)			
	HMCR = 0.5	HMCR = 0.6	HMCR = 0.7	HMCR = 0.8
PAR = 0.1	(21, 4.2147)	(26, 3.8124)	(29, 3.4721)	(27, 4.0112)
PAR = 0.2	(22, 4.1457)	(35, 2.0974)	(36, 2.1474)	(31, 3.3784)
PAR = 0.3	(21, 4.5714)	(38, 1.1124)	**(40, 0.8514)**	(35, 2.0053)
PAR = 0.3	(23, 4.0781)	(34, 2.0078)	(37, 2.0012)	(31, 3.4079)

**Table 2:** HS-RANSAC estimator parameters.

HMS	HMCR	PAR	BW_max_	BW_min_	NI	*λ*	Th
50	0.7	0.3	10	1	950	0.001	5

**Table 3:** Inlier detection comparison in terms of the detection rate (DR), the error (*E*_*r*_), and the number of function evaluations (NFE) for standard RANSAC [14], the MLESAC [20], the SIMFIT method [21], the projection-pursuit algorithm [22], the TSSE [23], the PSO algorithm (PSO-RANSAC) [54], and the proposed HS-RANSAC approach, considering the four test images shown in Figures 5, 6, 7, and 8.

Image	Method	Detected inliers (NofI)	Missing	False alarms	DR (%)	*E*_*r*_	NFE
(A) Total number of inliers (86)	Standard RANSAC	41	45	21	47.7	4.75	876
	MLESAC	55	31	14	63.9	3.11	852
	SIMFIT	62	24	11	72.0	2.98	842
	Projection-pursuit	58	28	12	67.4	3.53	798
	TSSE	48	38	14	55.8	3.42	815
	PSO-RANSAC	75	11	8	87.2	1.68	491
	HS-RANSAC	82	4	5	95.3	0.88	396

(B) Total number of inliers (72)	Standard RANSAC	32	40	18	44.4	3.98	765
	MLESAC	40	32	14	55.5	3.43	825
	SIMFIT	58	14	8	80.5	2.87	891
	Projection-pursuit	47	25	12	65.2	3.12	759
	TSSE	43	29	16	59.7	3.47	786
	PSO-RANSAC	63	9	5	87.5	1.51	374
	HS-RANSAC	70	2	3	97.2	0.79	328

(C) Total number of inliers (56)	Standard RANSAC	24	32	15	42.8	2.96	689
	MLESAC	27	29	11	48.2	2.41	628
	SIMFIT	42	14	9	75.0	1.98	724
	Projection-pursuit	37	19	13	66.0	2.85	754
	TSSE	32	24	14	57.1	2.74	776
	PSO-RANSAC	48	8	9	85.7	0.94	349
	HS-RANSAC	53	3	5	94.6	0.25	272

(D) Total number of inliers (122)	Standard RANSAC	62	60	22	50.8	4.02	832
	MLESAC	77	45	18	63.1	3.41	924
	SIMFIT	90	32	13	73.7	2.86	845
	Projection-pursuit	75	47	19	61.4	3.52	914
	TSSE	76	46	21	62.2	3.73	887
	PSO-RANSAC	110	12	10	90.1	1.41	427
	HS-RANSAC	115	7	5	94.2	0.51	338
